# Evaluation of Targeted Delivery to the Brain Using Magnetic Immunoliposomes and Magnetic Force

**DOI:** 10.3390/ma12213576

**Published:** 2019-10-31

**Authors:** Louiza Bohn Thomsen, Thomas Linemann, Svend Birkelund, Gitte Abildgaard Tarp, Torben Moos

**Affiliations:** 1 Laboratory of Neurobiology, Biomedicine Group, Department of Health Science and Technology, Aalborg University, 9220 Aalborg East, Denmark; tl@linemann.com (T.L.); Gitte.A.Arendt@hotmail.com (G.A.T.); 2 Laboratory of Medical Mass Spectrometry, Biomedicine Group, Department of Health Science and Technology, Aalborg University, 9220 Aalborg East, Denmark; sbirkelund@hst.aau.dk

**Keywords:** blood-brain barrier, drug delivery, in situ brain perfusion, magnetic nanoparticles, magnetic liposomes, magnetic immunoliposomes, OX26

## Abstract

Magnetic nanoparticles have great prospects for drug delivery purposes, as they can be designed with various surface coatings and conjugated with drugs and targeting moieties. They also have a unique potential for precise delivery when guided by magnetic force. The blood-brain barrier (BBB) denotes the interface between the blood and brain parenchyma and hinders the majority of drugs from entering the brain. Red fluorescent magnetic nanoparticles were encapsulated in liposomes and conjugated to antibodies targeting the rat transferrin receptor (OX26) to form magnetic immunoliposomes. These magnetic immunoliposomes enhanced the uptake by rat brain capillary endothelial cells (BCECs) in vitro. In situ brain perfusion in young rats high in the endogenous expression of transferrin receptors by BCECs, revealed enhanced uptake of magnetic immunoliposomes when compared to naked magnetic nanoparticles or non-targeted magnetic liposomes. When applying the external magnetic force, the magnetic nanoparticles were detected in the brain parenchyma, suggesting transport across the BBB. Ultrastructural examination of the immunoliposomes, unfortunately, was unable to confirm a complete encapsulation of all naked nanoparticles within the liposomes, suggesting that the data on the brain could derive from particles being released from the liposomes under influence of external magnetic force; hence hypothesizes on external magnetic force as a qualifier for dragging targeted magnetic immunoliposomes through the BBB. In conclusion, our results suggest that transport of magnetic nanoparticles present in BCECs by targeted delivery to the transferrin receptor may undergo further transport into the brain when applying magnetic force. While magnetic immunoliposomes are targetable to BCECs, their design to enable further transport across the BBB when applying external magnetic force needs further improvement.

## 1. Introduction

Nanotechnology enables the design of nanoparticles for numerous medical purposes; e.g., liposomes at the nanoscale are amenable for therapy in treatment of cancer and chronic diseases affecting the nervous system [1,2,3]. The magnetic liposomes consisting of liposomes shielding magnetic nanoparticles in a lipid bilayer, denote a new drug carrier that allows the magnetic guidance of nanoparticles by an external magnet. These liposomes can also be designed to contain polyethylene glycol (PEG)-conjugated phospholipids (PEGylated phospholipids) to increase the plasma half-life [4,5]. In addition, these PEGylated phospholipids can be functionalized by conjugating a targeting ligand or antibody [4,5,6,7].

Drug delivery systems targeting endogenous receptors of the brain are now considered one of the most promising approaches towards transporting therapeutics into the brain [8,9]. The delivery systems are targeted to nutrient transporters like transferrin receptors, CD98, or insulin receptors, which are all expressed at the blood-brain barrier (BBB) [8,9,10,11]. The use of magnetic liposomes may carry the potential for such a purpose by means of antibody-targeting to these endogenous molecules [12]. The BBB is formed by brain capillary endothelial cells (BCECs) held closely together by tight junctions, which restricts the paracellular transport of therapeutics [10,13]. Thus, targeted therapeutics can only access the brain parenchyma either because they are highly lipophilic or because they are able to utilize the naturally occurring transporters leading to intracellular transport through BCECs. Larger molecules otherwise unable to pass the BBB only can access the brain by targeted approaches [5,6,7,12].

For targeted approaches to access the brain, the transferrin receptor is of particular interest because the BCECs are abundant in transferrin receptors, unlike the vascular endothelium of the remainder of the body [6,9,14,15,16,17,18,19]. Therefore, anti-transferrin receptor antibodies conjugated to a variety of drug delivery systems have been thoroughly investigated for delivering therapeutics directly from the blood to the brain [16,17,18]. The transferrin receptor internalizes the antibody upon binding at the luminal surface of the BCECs but the subsequent transport has been subject of debate and controversy. Intravenous injection of anti-transferrin receptor antibodies reveals a substantial accumulation of antibodies in BCECs. However, consistent with data from recent studies by other groups, we never encountered convincing evidence of the transcytosis of high-affinity antibodies via the transferrin receptor [7,17,18,20,21,22,23,24,25]. 

When applying an external magnetic force for the delivery of magnetic nanoparticles to the brain, the particles will be dragged through BCECs and further into the brain tissue. Investigations of the impact and safety of this motion of particles through BCECs or neurons have shown no cytotoxic effects, no disruption of nerve fibers, and no influence on neuronal circuit function [26,27,28,29,30,31,32].

As use of an external magnetic field may prove a feasible method to facilitate the movement of nanoparticles into the brain [26,28,33,34], we tested the hypothesis that the application of an external magnetic field enhances the passage of magnetic liposomes conjugated to antibodies raised against the transferrin receptor. Conjugation of anti-transferrin antibodies to magnetic liposomes is the most likely option to increase the uptake by the BCECs, but will not necessarily lead to increased transcytosis. Therefore, we hypothesized that conjugation of an anti-transferrin antibody to magnetic liposomes would increase the uptake by the BCECs and that application of an external magnetic force would further increase the transport of magnetic liposomes into the brain.

Accordingly, we developed a magnetic responsive drug delivery system composed of magnetic Fe_3_O_4_ nanoparticles enveloped in a PEGylated bilayer to form magnetic liposomes. The magnetic liposomes were made targetable to the transferrin receptor by conjugation to antibodies against the rat transferrin receptor (OX26), resulting in the formation of magnetic immunoliposomes; i.e., magnetic OX26 immunoliposomes. The capability of the magnetic nanoparticles to accumulate within BCECs was studied in vitro. Furthermore, the capability of the magnetic OX26 immunoliposomes to cross the BBB was tested using in situ brain perfusion in 16-day old rats. These young rats express substantially higher levels of transferrin receptors than their adult counterparts [17].

## 2. Materials and Methods 

### 2.1. Production, Purification, and Specificity Control of Anti-Transferrin Receptor (OX26) Monoclonal Antibodies

OX26 synthesizing hybridoma cells (Sigma-Aldrich, Saint Louis, MO, USA) were cultured in CELLine™ CL1000 Bioreactor (BD Biosciences™) in ProDoma™ medium (Lonza, Basel, Switzerland) supplemented with 1% fetal calf serum (FCS). Supernatants containing antibodies were harvested weekly, clarified by centrifugation at 2000 rpm for 5 min, and stored at −83 °C until purification. 

The OX26 monoclonal antibodies (OX26-MAbs) were purified using a GE-healthcare, XK-column 16, packed with protein G coupled Sepharose gel dissolved in a binding buffer made up of 1 M glycine and 0.15 M NaCl at pH 8.5. The antibody-containing harvest was filtered through a 0.22 µm filter to remove any remaining bulk proteins, dead cell debris, and particulate matter. Supernatants were mixed 1:1 v/v with binding buffer. OX26-MAbs were eluted by running elution buffer consisting of 0.1 M glycine at pH 2.7 through the column, collected in 2 mL fractions, and neutralized by adding 10% v/v 1 M Tris-HCl, pH 9.0. Absorbance at 280 nm was measured by spectrophotometer (Thermo Scientific) to select the elution fractions, containing the OX26-MAbs. For the dialysis of selected elution fractions we used dialysis tubing, Spectra/Por 1 with MWCO of 6–8 kDa (Spectrumlabs). Samples were dialyzed in triplicates against HEPES (4-(2-hydroxyethyl)-1-piperazineethanesulfonic acid)-buffer (136 mM NaCl, 10 mM HEPES, and 1 mM EDTA) in v/v ratio of 1:100.

Cells transfected with rat transferrin receptor were examined for their specificity of their OX26-MAbs. Plasmid DNA (pCMV-ratTFRC-10his (Sino Biological Inc., Beijing, China)) encoding Rattus norvegicus transferrin receptor (NM_022712) was prepared from *Escherichia coli* DH5α. HeLa cells were transfected with 1 μg plasmid DNA using TurboFect Transfection Reagent for in vitro transfection (Thermo Scientific, Waltham, MA, USA) according to manufacturer’s instructions. Non-transfected cells were used as negative control. After 24 h, the cells were fixed with 4% paraformaldehyde for 10 min, permeabilized with 0.2% Triton X-100 in PBS, and blocked for non-specific binding of antibodies with 0.05% bovine serum albumin (BSA) in PBS [35]. Five μg/mL of purified OX26-MAb in PBS containing 0.05% BSA was incubated for 30 min at 37 °C and followed by 3 washes in PBS before the adding of FITC-conjugated goat anti-mouse IgG (Jackson Immuno Research, West Grove, PA, USA) diluted 1:100 for 30 min at 37 °C. The cells were washed in PBS, and cell nuclei were stained with 1 μM To-Pro-3 dye (Life technologies, Thermo Fisher Scientific, Roskilde, Denmark) in PBS for 10 min.

### 2.2. OX26-MAbs’ Binding to Rat Brain Endothelial Cells

The affinity of OX26-MAbs was also examined in immortalized rat brain endothelial cells (RBE4s) that avidly express transferrin receptors [6]. RBE4 cells were cultured in growth medium made up of 50% Alpha-MEM with Glutamax-1 (Gibco, Thermo Scientific, Waltham, MA, USA) and 50% HAM’s F-10 with Glutamax-1 (Gibco, Thermo Scientific, Waltham, MA, USA) with supplementary 10% FCS, 1% penicillin-streptomycin (Gibco, Thermo Scientific, Waltham, MA, USA), 300 μg/mL Geneticin Sulfate (Acros Organics, Fisher Scientific, Hampton, NH, USA), and 1 ng/mL basic fibroblast growth factor (Invitrogen, Carlsbad, CA, USA). Immunocytochemistry on RBE4 cells was performed using OX26-MAbs and commercially available mouse anti-rat transferrin receptor CD71 antibodies (Serotec, Oxford, UK). The RBE4 cells were fixed in 4% paraformaldehyde for 15 min, blocked for non-specific binding of antibodies using 0.05% bovine serum albumin (BSA) in PBS, and followed the by addition of the primary antibody (stock concentration 1 mg/mL) in dilutions of 1:100, 1:200, and 1:400 for 1 h at 37 °C. Next, biotinylated goat anti-mouse antibody (DAKO) was added (1:200) for 30 min followed by streptavidin Alexa Fluor^®^ 488 (Invitrogen, Oxford, UK) (1:200) for 30 min. Nuclei were counterstained with 4’,6-diamidino-2-phenyindole (DAPI) in a concentration of 2 µg/mL and placed under cover slips with fluorescence mounting medium (DAKO). The cells were washed in PBS in triplicate between each step.

The affinity of OX26-MAbs towards BCECs was also tested in vivo by injecting a bolus of 200 µL containing 100–300 µg OX26-MAbs in HEPES-buffer intravenously in 16 postnatal (P) days Wistar rats. After two hours, the rats were deeply anesthetized by a subcutaneous injection of 0.5 mL/10 g body weight of Hypnorm/Dormicum (Fentanyl/Fluanisone mixed with Midazolam) and fixed by vascular perfusion [7]. The brains were treated as described below. The brain from an un-injected rat served as a negative control.

### 2.3. Preparation of Magnetic Liposomes 

**Figure 1:**
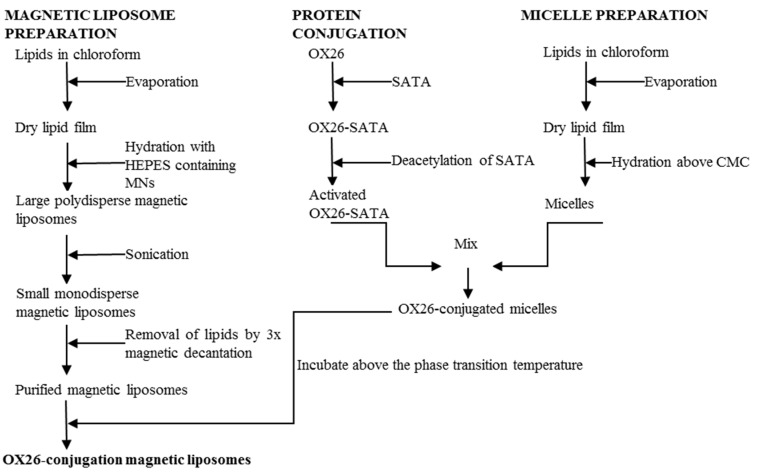
Overview of magnetic OX26 immunoliposome synthesis that consists of three separate processes. (**i**) Preparation of magnetic liposomes by lipid film hydration and sonication; (**ii**) modification of OX26-MAbs with SATA, plus preparation of micelles containing maleimide-functionalized lipids; (**iii**) conjugation of micelles with activated SATA-modified OX26-MAbs, and those being inserted into preformed magnetic liposomes to yield magnetic OX26 immunoliposomes. CMC: Critical micelle concentration; MNs: Magnetic nanoparticle.

The lipid-encapsulation process of magnetic nanoparticles with red fluorescent dye (Chemicell, Berlin, Germany) was based the previously-described method (Figure 1) [36]. The magnetic nanoparticles were paramagnetic, meaning the particles could be magnetized by subjection to an external magnetic field. The net magnetic moment drops to zero when the external magnetic field is removed; hence, the magnetic nanoparticles do not retain any net magnetic properties without an external magnetic field. A mixture of L-α-phosphatidylcholine (Soy PC) (Avanti Polar lipids, Alabaster, AL, USA), dimethyldioctadecylammonium bromide (DDAB) (Sigma), and 1,2-dipalmitoyl-sn-glycero-3-phosphoethanolamine-*N*-[methoxy(polyethylene glycol)-2000] (mPEG2000-PE), dissolved in chloroform, was prepared in molar ratio of 37:60:3. This suspension was dried into a thin lipid film under a continuous nitrogen gas stream until no solvent was visible. The resulting film was rehydrated in HEPES-buffer containing magnetic nanoparticles in w/w ratio of 1:10 (magnetic liposomes/solid lipid) and vortexed. The mixture was placed under constant agitation at ambient temperature for 1 h and then extensively sonicated for 1.5 h to dissolve any aggregates and promote the formation of unilamellar liposomes. Excess lipid was removed by triple magnetic decantation using DynaMAG™-2 magnet (Invitrogen, Oxford, UK), followed by resuspension in HEPES buffer.

### 2.4. Synthesis of OX26-MAb-Conjugated Magnetic Immunotoliposomes Using the SATA Method

Magnetic OX26 immunoliposomes were produced by transferring OX26 coupled to MAL-PEG2000-DSPE into magnetic liposomes (Figure 1) [37]. The micelles were prepared by mixing 1,2-distearoyl-sn-glycero-3-phosphoethanolamine-*N*-[maleimide(polyethyleneglycol)-2000] (DSPE-PEG2000-maleimide) (Avanti Polar Lipids, Alabaster, AL, USA) and mPEG2000-PE in a molar ratio of 4:1. Lipids dissolved in chloroform were dried into lipid film under continuous nitrogen. The resulting lipid film was rehydrated in HEPES-buffer preheated to 65 °C at a concentration above the critical micelle concentration. Micelles were formed by heating to 65 °C under continuous agitation for 1 h at 1400 rpm in a Thermomixer (Eppendorf Nordic, Hørsholm, Denmark).

OX26-MAbs were thiolated by dissolving 10 mg SATA/mL (*N*-succinimidyl-*S*-acetylthioacetate) in *N,N*-dimethylformamide and mixing with OX26-solution in molar ratio of SATA:OX26 8:1. The mixture was incubated for 45 min at ambient temperature during continuous rotation. Free SATA was removed by centrifugation in Vivaspin 6 ultrafiltration with 50 kDa cut-off (GE healthcare). Sulfhydryl groups were activated by adding freshly hydroxylamine solution (0.5 M hydroxylamine HCl, 0.5 M HEPES, and 25 mM EDTA) to OX26-MAbs resuspended in HEPES-buffer solution in 1:10 v/v ratio and incubated for 1 h at room temperature during continuous rotation. OX26-MAbs were conjugated to micelles by mixing deacetylated OX26-MAbs with micelles in molar ratio of 1:10 for OX26:DSPE-PEG2000-maleimide and incubating the solution for 2 h at room temperature and overnight at 4 °C on an agitator table. OX26-conjugated micelles were mixed with magnetic liposomes in w/w ratio of magnetic liposomes to micelle phospholipids of 1:1.25. The suspension was heated to 60°C for 1 h during continuous rotation at 1400 rpm in Thermomixer and cooled to room temperature. Non-bound OX26 and micelles were removed from the sample by magnetic decantation. The resulting magnetic OX26 immunoliposomes were finally resuspended in 1.5 mL HEPES-buffer.

### 2.5. Determination of the Particle Concentration after Synthesis

Determination of magnetic liposomes’ and magnetic OX26 immunoliposomes’ concentrations relied on their iron content based on the Perl’s Prussian Blue reaction, which allows for blue-staining of iron-containing solutions when digested in hydrochloric acid and potassium ferrocyanide [38]. Ferrocyanide reacts with ferric chloride to form ferric ferrocyanide with an absorption around 700 nm [38]. A standard curve was established from the magnetic liposomes provided by diluting 1 to 12 µg of magnetic liposomes in HEPES-buffer. Two hundred µL 5M HCl was added for 4 h at 80 °C in Thermomixer at 450 rpm. Finally, 100 µL of 5% aqueous solution of ferrocyanide was incubated at room temperature for 15 min. The absorbance was measured at 650 nm. Three samples of magnetic liposomes and magnetic OX26 immunoliposomes were treated similarly. Concentrations of magnetic liposomes and magnetic OX26 immunoliposomes were determined from the standard curve.

### 2.6. Determination of the Size and Zeta-Potential, and Analysis with Transmission Electron Microscopy 

Mean particle size and charge were assessed in Zetasizer Nano ZS (Malvern Panalytical, Malvern, UK) by dynamic light scattering and zeta potential determined by laser doppler electrophoresis. Measurements were performed on three separate samples suspended in HEPES-buffer: Data were analyzed using Malvern Zetasizer Software, version 6.2. To assess the size stability, a fraction of the magnetic liposomes was kept at 4 °C over a period of 6 weeks. The hydrodynamic size was measured once a week.

5–10 μL of the solution with magnetic nanoparticles or magnetic OX26 immunoliposomes were loaded on glow-discharged, carbon-coated copper grids. The excess solution was sucked away and the samples were air-dried. A 1% uranyl acetate solution was added and the samples were left to air-dry. The samples were visualized using a Fei Tecnai Spirit TEM microscope (Hillsboro, OR, USA) operated at 120 kV.

### 2.7. Determination of the Antibody Concentration on the Nanoparticles

The antibody concentration was determined with RC DC Protein Assay (Bio-Rad) based on the Lowry assay and measured by absorbance at 750 nm [39]. A standard curve was made with dilutions of human IgG (Sigma Aldrich, Saint Louis, MO, USA) in the range of 0.2–1.5 mg/mL. For determination of the OX26 concentration of the magnetic OX26 immunoliposomes, the absorbance of equivalent amounts of magnetic liposomes was subtracted to correct the measured absorbance of magnetic OX26 immunoliposomes.

### 2.8. Binding and Uptake Magnetic Nanoparticles, Magnetic Liposomes, and OX26-Magnetic Immunoliposomes

To RBE4 cells seeded in collagen-coated Permanox^®^ chamber slides at a density of 30,000 cells/cm^2^ and cultured for 24 h, 1 µg of magnetic nanoparticles, magnetic liposomes, or magnetic OX26 immunoliposomes were added to separate wells and they were incubated for 3 h. Unbound particles were removed by three washes with PBS, followed by fixation for 15 min with 4% paraformaldehyde, and finally, the addition of 2 µg/mL DAPI.

Quantization on particle uptake was assessed by computed mean fluorescence intensity of 30–50 individual measurements in each group analyzed in ImageJ version 1.45 s using the free hand selection tool (NIH Imaging: http://rsb.info.nih.gov/ij) [35,40,41]. Comparison between mean fluorescence intensities of the uptake of magnetic nanoparticles, magnetic liposomes, and magnetic OX26 immunoliposomes after 3 hours was obtained by measuring the total area of each cell with a fluorescence signal. Median fluorescence intensity was calculated in SPSS. Since variances could not be assumed, Mann–Whitney U tests were used to determine significance between groups (*p* < 0.05).

### 2.9. Cell Viability

RBE4 cells were seeded in collagen-coated 12 well culture plates and cultured for three days until magnetic nanoparticles, magnetic liposomes, or magnetic OX26 immunoliposomes were added in a concentration of 50 μg/cm^2^ at experimental day 0. After 8 hours of incubation, the particles were removed, and the cells in 4 wells were trypisinized and stained with 0.2% trypan blue. Both viable and non-viable cells were then counted, and the cell viability was determined. Thereafter, the viability was also determined at experimental day 1, 3, 5, 7, and 11. A two-way ANOVA followed by Tukey’s multiple comparisons test was performed to test if there was a difference between the viability of all the non-treated cells and the treated cells. 

### 2.10. In Situ Brain Perfusion

In situ brain perfusion was performed on P16 Wistar rats as previously described [7]. Daily care of the rat until the experiments was handled at the animal facility at Sygehus Nord, Aalborg. The rats were deeply anesthetized by subcutaneous injection of 0.5 mL/10 g body weight of Hypnorm/Dormicum and perfusion was performed transcardially by insertion of a G23 needle (Terumo, Somerset, PA, USA) connected to the peristaltic tubing in the left ventricle. The circulatory system was initially perfused for 30–45 seconds with isotonic saline containing 100 IU/mL heparin (LeoPharma, Malmö, Sweeden), and the perfusion then continued at 5 mL/min for 15 min with an M199 medium free of plasma proteins (Gibco, Thermo Scientific, Waltham, MA, USA) with added nanoparticles. The perfusion time was limited to 15 minutes, as longer perfusion periods can cause hypoxia and compromise the BBB integrity [7,16,42]. The M199 medium was kept at 37 °C in a water bath and continuously oxygenated by bubbling 100% O_2_. There were two variables in the perfusion protocol; i.e., particle type and presence or absence of an extracranially placed magnet. The rats received nanoparticles based on calculations that ensured a dose of 10 µg iron/g bodyweight independent of particle type. The magnet used for the experiments was a 1.40–1.46 Tesla neodymium disc magnet placed extracranially in intimate contact with the superior portion of the rat’s scalp during the entire in situ brain perfusion. The number of rats perfused with an external magnet were *n* = 4 for magnetic nanoparticles, *n* = 4 for magnetic liposomes, and *n* = 8 for magnetic OX26 immunoliposomes. An equal number was perfused with either compound without the magnet. The experiments ended with a quick wash of the circulatory system with isotonic saline, followed with 4% paraformaldehyde for 10 min, which ensured that brain capillaries were emptied for nanoparticles [7]. Dissected brains were fixed in 4% paraformaldehyde for 24 h, immersed in sucrose, and cut into 30–35 µm serial coronal sections at −25–30 °C on a cryostat [7].

### 2.11. Immunohistochemistry 

Free-floating immunohistochemistry was performed to detect the intravenously injected OX26 [7]. Sections were incubated overnight at 4 °C with donkey anti-mouse conjugated with Alexa Fluor 555 (1:200). To localize the basal lamina of brain capillaries, sections were incubated with polyclonal rabbit anti-laminin (DAKO) (1:100) overnight [7]. The next day, the sections were incubated with goat-anti-rabbit Alexa 488 (1:200).

### 2.12. Fluorescence and Confocal Microscopy

Fluorescence microscopy was carried out on inverted microscopes Axiovert 200M and Axio Observer.Z1 (Carl Zeiss, Birkerød, Denmark). Images were captured with a highly sensitive monochrome camera AxioCam MRm using Axiovision version 4.8.2 imaging software. A Leica confocal SP5 microscope with an HCX PL APO 100x /1.47 oil objective, equipped with an argon multiline laser at 488 nm and an HeNe laser at 543 nm was sequentially used to scan the sections for the Z-stack (Leica, Brønshøj, Denmark).

## 3. Results

### 3.1. Syntheses and Specificities of OX26-MABs

**Figure 2:**
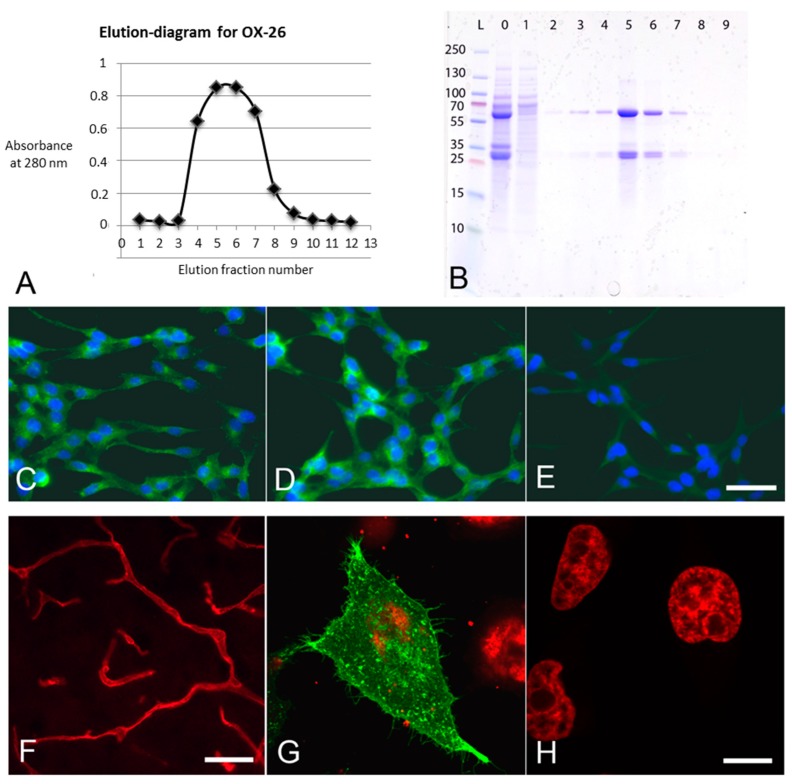
Specificity of OX26-monoclonal antibodies (MAbs). (**A**) Elution diagram of collected OX-MAbs. X-axis: Elution fraction number; y-axis: Absorbance at 280 nm. (**B**) Coomasie Blue-stained SDS gel. Lane L, protein ladder (kDa). Lane 0, sample of antibody suspension before running on affinity column. Lane 1, sample from flow-through when loading affinity column with antibody suspension. Lane 2, sample of flow-through immediately before elution of antibodies. Lanes 3–9, elution fractions containing the expected 25 and 55 kDa bands representing components of OX26-MAbs. Lanes 5–7 contain the majority of OX26-MAbs corresponding to the high absorbance in A. The absence of 25 and 55 kDa bands in lane 1 indicates that OX26-MAbs were well adsorbed to the affinity column. (**C**–**E**) Merged images captured from RBE4 cells labeled with OX26-MAbs (**C**), commercial anti-CD71 (**D**), or secondary antibody alone (**E**). (**F**) The detection of OX26 in brain sections following the intravenous administration of 300 µg OX26-MAbs. (**G**–**H**) HeLa cells transfected with cDNA encoding rat transferrin receptor protein. Detection of the rat transferrin receptor protein using OX26-MAbs using FITC-conjugated secondary antibody (green) in transfected cells (**G**) and non-transfected cells (**H**), indicative of the affinity of OX26-MAbs for the rat transferrin receptor. Cellular nuclei stained with To-Pro3 (red). Scale bars = 20 µm (**C**–**E**), 20 µm (**F**), and 10 µm (**G**–**H**).

Different fractions of OX26-MAbs collected during elution were used in order to create an elution diagram (Figure 2A). The purity of OX26-MAbs was analyzed by SDS PAGE. In Coomasie stains, the 25 and 55 kDa bands stood out, corresponding to the molecular weight of IgG light and heavy chains, respectively (Figure 2B). The OX26-MAbs were tested against commercially available anti-CD71 antibody, and both antibodies equally labeled transferrin receptors at the cell membrane and cytosol (Figure 2 C,D). No labeling was seen when these antibodies were omitted (Figure 2E). 

To determine the targeting potential of OX26-MAbs, immunohistochemistry was performed on brains from rats intravenously injected with OX26-MAbs (Figure 2F). The OX26-MAbs immunoreactivity revealed distinct labeling consistent with targeting, uptake, and transport into BCECs [7,14,15,16,17]. Sections from control brains without prior injection of OX26-MAbs were devoid of immunolabeling (not shown). To further evaluate the OX26-MAbs reactivity, the rat transferrin receptor was transiently expressed in human HeLa cells [43]. A strong labeling was seen within the cytoplasm of transfected cells (Figure 2G). The immunoreaction was seen as dots and vacuoles within the cytoplasm corresponding to the expected localization of the transferrin receptor [43]. Non-transfected HeLa cells used as negative controls were non-reactive when subjected to OX26-MAbs (Figure 2H).

### 3.2. Particle Characterization

The preparation of magnetic nanoparticles led to an increase in particle size from 117.17 ± 1.17 nm to 150.20 ± 2.44 nm (Table 1), accompanied by an increase in the zeta potential from −14.83 ± 0.42 mV to +15.66 ± 1.65 mV, which was indicative of encapsulation in a cationic lipid layer. As single phospholipid bilayers are roughly 5 nm thick, and a corona formed by PEG-2000 has an area of equal thickness, an apparent increase of 20 nm in total diameter was expected [44]. However the actual overall increase was 10 nm higher than expected, suggesting that encapsulation entrapped water along the particles or consisted of more lipid layers. The polydispersity index (PDI) increased from 0.17 to 0.23, indicative of a slightly more non-homogeneous sample while encapsulating magnetic nanoparticles inside liposomes. The size was stable for at least 5 weeks, but began to show signs of instability after 5 weeks of storage at 4 °C. (data not shown). 

After conjugating OX26-MAbs to magnetic liposomes, the particle size increased to 181.90 ± 6.32 nm, indicative of the significance of transferring OX26-MAbs to the lipid surface, since the length of one such antibody with a linker is approximately 15 nm [44]. The increase in PDI to 0.29 indicated low level of aggregates formed during the antibody transfer from micelles to magnetic liposomes, since the temperature during this step was above the phase transition temperature that allowed lipid membranes to fuse. The zeta potential of the magnetic OX26 immunoliposomes was reduced by approximately 20 mV, probably due to transfer of anionic DSPE-PEG2000-maleimide from micelles to magnetic liposomes. Incorporating anionic PEGylated phospholipids was previously reported to reduce zeta potentials of cationic magnetic liposomes [36]. The antibody density in the final OX26-magnetic immunoliposome batch was determined as ≈85 antibodies per OX26-conjugated magnetic liposomes based on the assumptions that the manufacturer’s predicted particle number of magnetic nanoparticles per gram was 1.8 × 10^15^/g; that 10% of OX26-MAbs are associated with the particles; and OX26 weighs 160,000 g/mol. Based on measurements of the OX26 protein concentration before and after conjugation to magnetic liposomes, it was estimated that approximately 95% of the protein was present in the fraction containing magnetic liposomes, indicating a very high incorporation efficiency of the OX26-MAbs. 

The size of the magnetic nanoparticles was examined using TEM and revealed diameters around 100 nm, but some smaller magnetic nanoparticles could also be observed with a diameter of approximately 50 nm (Figure S1A). Magnetic nanoparticles embedded in OX26-liposomes were also visualized by TEM (Figure S1B). magnetic OX26 immunoliposomes could be observed containing a single, or in a few instances, two encapsulated magnetic nanoparticles. A fraction of immune-liposomes devoid magnetic nanoparticles was, however, also detected. Furthermore, a small amount of un-encapsulated magnetic nanoparticles was observed. The size of the magnetic OX26 immunoliposomes was around 3–400 nm in diameter in average, but they varied in the range of approximately 200 to 500 nm in diameter. 

### 3.3. Cellular Uptake of Nanoparticles In Vitro

**Figure 3:**
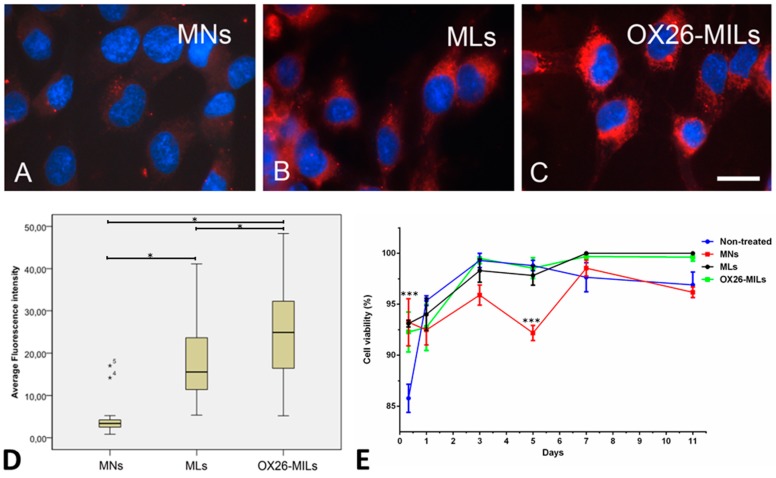
The uptake of red fluorescent nanoparticles in RBE4 cells, and cell viability. Cells were incubated with 1 µg of magnetic nanoparticles (MNs), magnetic liposomes (MLs), or OX26-immunomagnetic liposomes (OX26-MLs). (**A**) Uptake is virtually absent in cells incubated with magnetic nanoparticles (MNs) without a liposome coat. Magnetic liposomes (MLs) (**B**) and magnetic OX26 immunoliposomes (OX26-MLs) (**C**) exhibit prominent fluorescence inside cells, indicative of uptake. Scale bar = 10 µm. (**D**) Quantitative estimate of the fluorescence emitted by RBE4 cells after the addition of magnetic nanoparticles (MNs), magnetic liposomes (MLs), or magnetic OX26 immunoliposomes (OX26-MILs) based on computed mean fluorescence intensity of 30–50 individual measurements in each group. The three groups are all significantly different (*p* ≤ 0.05), with MNs displaying the lowest intensity and OX26-MLs the highest. Outlier values are marked with asterisks numbered 4 and 5, due to their falling outside the definition as being values between 1.5 and three box lengths from the upper and lower edge of the box, respectively. (**E**) Cell viability of non-treated RBE4 cells or RBE4 cells treated for 8 h of incubation with MNs, MLs, and OX26-MILs. After 8 h, the viability of non-treated cells was significantly lower (*p* ≤ 0.001) than all the treated cells. At day five, the viability of MNs was significantly lower (*p* ≤ 0.001) than the non-treated cells and cells with MLs and OX26-MILs.

RBE4 cells revealed a very low uptake of unmodified nanoparticles. By comparison, both magnetic liposomes and magnetic OX26 immunoliposomes exhibited improved binding and uptake by RBE4 cells (Figure 3). The particles were confined to the cytosol and revealed a distinct perinuclear accumulation that corresponds to reports on nanoparticle distribution in endothelial cells [36]. As fixation with paraformaldehyde fixation may lead to redistribution of fluorophores within cells [44]; therefore, we also examined the distribution of magnetic liposomes before and after fixation. We also examined whether prolonging the exposure time of the cells for the magnetic liposomes would change the subcellular distribution of the magnetic liposomes, which could indicate that the cells had degraded the magnetic liposomes. However, we did not find signs of a significant redistribution of magnetic liposomes after fixation (Figure S2), and we did not observe any changes in the subcellular distribution of the RBE4 cells when incubating the cells with magnetic liposomes for 3, 12, or 48 h. Furthermore, the viability of RBE4 cells incubated with magnetic liposomes and magnetic OX26 immunoliposomes did not significantly differ from non-treated cells for up to 11 days after treatment. Magnetic nanoparticles were significantly different (*p* ≤ 0.001) from the non-treated, magnetic liposomes and magnetic OX26 immunoliposomes at day 5, but the cells recovered, and no difference was detected on days 7 or 11.

To provide quantification of the particle uptake, individual cells were marked, and the mean fluorescence intensity computed. Data were displayed as boxplots based on individual measurements of 30–50 in each of the three groups. The Kruskall–Wallis test revealed that the groups were significantly different (*p* < 0.05). Mann–Whitney U tests were run between different groups. Both magnetic liposomes and magnetic OX26 immunoliposomes exhibited fluorescence greater than those of magnetic nanoparticles (*p* < 0.05) (Figure 3). The uptake of magnetic liposomes compared to pure magnetic nanoparticles was increased 4.5-fold, which is consistent with higher uptake of cationic liposomes in endothelial cells [44]. Further, an increase in uptake was seen when OX26-MAbs were conjugated to magnetic liposomes, as demonstrated by a 7.3-fold increase compared to pure magnetic nanoparticles, and a 1.6-fold increase compared to magnetic liposomes.

### 3.4. In Situ Brain Perfusion

**Figure 4:**
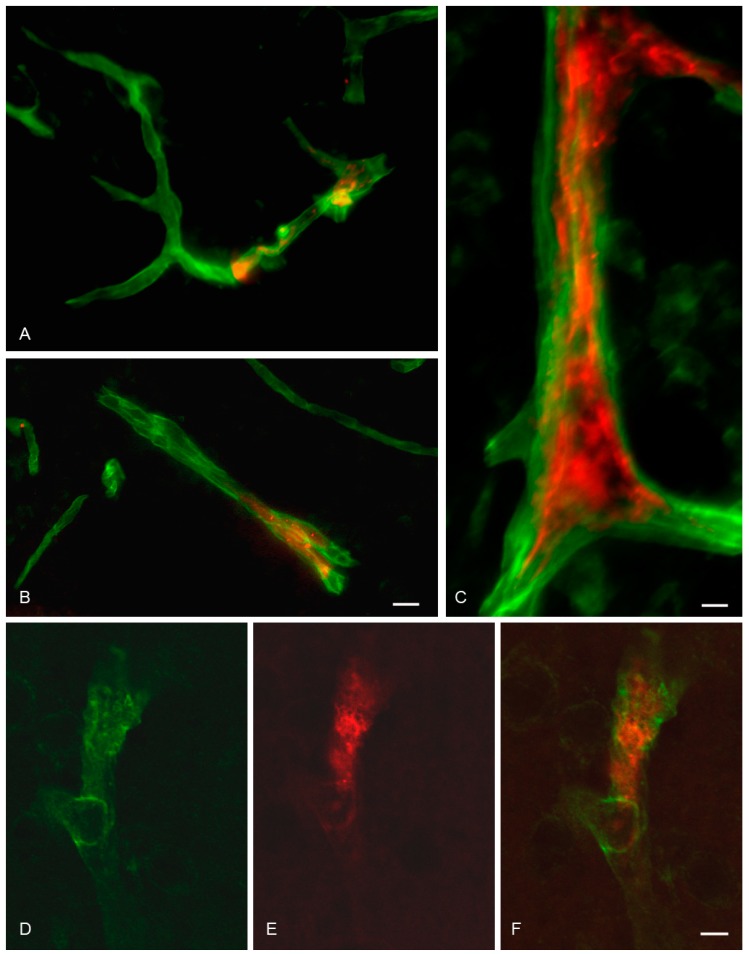
Brain distribution of magnetic OX26 immunoliposomes following in situ perfusion without application of external magnetic force. Images are from rats which received 10 µg/g bodyweight of magnetic OX26 immunoliposomes. (**A**–**C**) Magnetic OX26 immunoliposomes (red) present inside brain capillaries do not distribute past laminin (green) which marks the basal membrane of brain capillaries. (**D**–**F**) Co-detection of laminin (green) and magnetic OX26 immunoliposomes (red) using confocal imaging. The pictures show a Z-stack generated from serial analyses of a single brain capillary. (**F**) Merging the two fluorophores reveals that magnetic OX26 immunoliposomes are confined to the interior of the brain capillary. Scale bars = 20 µm (**A**,**B**), 10 µm (C), and 5 µm (**D**–**F**).

When perfused with magnetic liposomes or naked magnetic nanoparticles, BCECs in the brain parenchyma were only labeled vaguely. The was no apparent difference in the distribution between brain sections from rats exposed to or without magnetic field exposure (not shown). In contrast, brain perfusion with magnetic OX26 immunoliposomes revealed a prominent labeling of the vascular bed (Figure 4). It should be noted that the red fluorescent labeling was only associated with the magnetic nanoparticles, and detection of magnetic OX26 immunoliposomes was, therefore, solely based on detection of the magnetic nanoparticles carried by the magnetic OX26 immunoliposomes. Therefore, we can only vouch for the movement of magnetic nanoparticles, and we can merely speculate whether they are enveloped in the magnetic OX26 immunoliposomes in all situations, especially after the application of a magnetic force where they are likely to be dragged out of the liposomes. To avoid confusion, we will still refer to detection of magnetic OX26 immunoliposomes, even though it was only the magnetic nanoparticles which were detected. The brain capillaries were labeled even without being exposed to the magnetic field, indicating that the higher uptake of magnetic OX26 immunoliposomes when compared to magnetic liposomes could be attributed to uptake by BCECs due to binding to the transferrin receptor. The co-detection with laminin that marks the basal membrane revealed that magnetic OX26 immunoliposomes were confined to the interior of the BCECs (Figure 4). 

**Figure 5:**
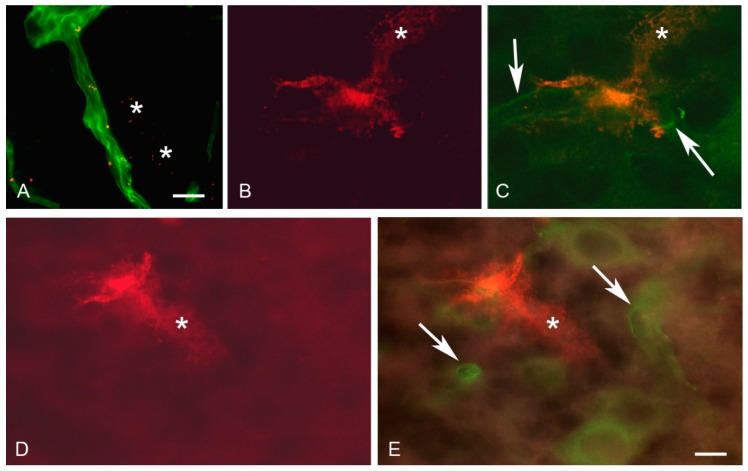
Brain distribution of magnetic OX26 immunoliposomes following in situ perfusion and the application of external magnetic force. Images are from rats which received 10 µg/g bodyweight of magnetic OX26 immunoliposomes while being subjected to an externally placed magnet. (**A**) Magnetic OX26 immunoliposomes (red) are present both inside and beyond (asterisks) an intraparenchymal brain capillary, with its boundaries indicated by laminin (green). (**B**,**C**) Detection of magnetic OX26 immunoliposomes’ (red) leakage from a brain capillary using confocal imaging. (**B**) Single detection of magnetic OX26 immunoliposomes. (**C**) Merging magnetic OX26 immunoliposomes (red) with laminin (green, arrows) reveals the presence of magnetic OX26 immunoliposomes beyond the boundaries of the brain capillary (**B**,**C**, asterisks ). (**D**,**E**) Detection of magnetic OX26 immunoliposomes’ (red) leakage from a different brain capillary than those seen in B and C. The extravasated magnetic OX26 immunoliposomes (asterisks) do not co-distribute with laminin (green, arrows). Scale bars = 20 µm (**A**), 5µm (**B**–**E**).

When exposed to the magnetic field, magnetic OX26 immunoliposomes permeated the intraparenchymal capillaries in a distinct perivascular labeling unlike that of laminin (Figure 5). This distribution was principally different from what was observed in brains without exposure to the magnetic field, suggesting that magnetic OX26 immunoliposomes not only associated with the basal membrane but also distributed into the brain past the entire vascular component, indicative of transport through both BCECs and the basal membrane (Figure 5). Aggregation of the magnetic nanoparticles, magnetic liposomes, and magnetic OX26 immunoliposomes in the vessels was to some extent observed after brain perfusion (data not shown). 

## 4. Discussion

### 4.1. Methodological Considerations

The in vitro uptake studies proved that embedding magnetic nanoparticles into a cationic lipid bilayer led to a 4.5 fold increase in fluorescence intensity within the RBE4 cells, which is consistent with prior observations of facilitated uptake of cationic liposomes in endothelial cells [36]. The increased uptake of magnetic liposomes compared to magnetic nanoparticles was probably due to favorable interactions of cationic liposomes with the cell membrane and subsequent uptake by adsorptive endocytosis [27,36,45]. 

It was important to address whether OX26-MAbs mediated improved uptake of magnetic OX26 immunoliposomes. The initial studies in HELA and RBE4 cells proved that OX26-MAbs had affinity for the rat transferrin receptor, which was additionally confirmed by the fact that intravenous injection of purified OX26-MAbs revealed labelling of BCECs in vivo. The uptake of magnetic liposomes increased when conjugated with OX26-MAbs; a 7.3 fold increase compared to naked magnetic nanoparticles and a 1.6 fold increase compared to magnetic liposomes. This suggests that conjugating OX26 to liposomes containing magnetic nanoparticles could be a suitable way of improving the uptake into BCECs [46].

It is critical to design homogenous particles to enable sufficient biodistribution following intravascular injection. An acceptable PDI is <0.3, and particles should not exceed 1.4 µm to avoid embolus formation [47,48,49]. The particles used in the present study met these criteria with a mean hydrodynamic size lower than 200 nm for the three types of particles, and a PDI within an acceptable range <0.3. TEM, however, showed a larger mean size of 3–400 nm in diameter for the magnetic OX26 immunoliposomes, which may be due to differences in the measuring methods. The DLS measurements were made in HEPES buffer, but the particles were air-dried in TEM preparations. 

Aggregation of the magnetic particles was observed in the vessels after in situ brain perfusion. The aggregation of magnetic nanoparticles is a known phenomenon both in vitro and in vivo, often due to the surface interaction of the nanoparticles with, e.g., salts and proteins in the biological milieu or due to the magnetic field causing drift-dominated agglomeration [26,50]. We speculate that the aggregation observed in the present study could be due to the interaction of the biological fluids, temperature, the surface coat (starch) of the magnetic nanoparticles, the interactions of the magnetic nanoparticles after application of the magnetic force, or a combination thereof. Agglomeration of naked magnetic nanoparticles has no impact on neuronal functionality and does not exert damage on brain tissue after the application of an external magnet in mice [26]. Nonetheless, the aggregation could have a strong impact on the magnetic properties of the magnetic nanoparticles [50], and the risk of an embolus formation of aggregates is also of concern. These topics should be subjects for further investigations and improvements.

Using the in situ brain perfusion, we demonstrated a preferential accumulation of magnetic OX26 immunoliposomes in the BCECs of the P16 rats that both have a lower systemic blood volume than their adult counterparts, which results in a higher concentration of nanoparticles in the plasma per injection, and have higher expression and recycling rate of transferrin receptors by BCECs [17]. The in situ brain perfusion method enables complete control of the test compound concentrations and the perfusion fluid constituents made devoid of plasma proteins, which are factors important to control as they lower the risks of the degradation or aggregation of nanoparticles [1,2,34,51].

In the present study, we did not aim to target magnetic nanoparticle accumulation into a specific brain region for delivery aided by a magnetic force. We used a 1.40–1.46 Tesla neodymium disc magnet and for easy application; the magnet was placed extracranially directly on the superior portion of the rat’s scalp. Accordingly, the regions with the highest detectable amounts of magnetic nanoparticles outside the vessels were in the brain sections in near vicinity of the location where the magnet had been placed. If a specific brain region or, e.g., a tumor, is the main target of delivery, the strength of the magnet versus the distance from the scalp to the region of interest should of course be considered [26,34].

### 4.2. Uptake and Transport of OX26-Magnetic Immunoliposomes at the BBB

Transport of magnetic OX26 immunoliposomes across the BBB was not observed in rats without exposure to magnetic force. This observation does not correlate with previous studies indicating that non-magnetic OX26 conjugated to PEGylated liposomes could target the brain parenchyma [6,42,52,53,54]. However, whether OX26 conjugated PEGylated liposomes can pass the BBB and enter the brain parenchyma is still up for debate [23]. Several studies show that OX26 cannot facilitate the transport of drug carriers into the brain parenchyma, but will instead be retained in BCECs [7,17,20,55], supporting our findings. 

The magnetic nanoparticles were found outside the vessels when rats were subjected to a magnetic force during in situ perfusion with magnetic OX26 immunoliposomes, suggesting movement of magnetic nanoparticles through the BBB by transcellular transport. The notion of magnetic nanoparticles’ passage through BCECs by means of transcellular rather than paracellular transport gains further support in that RBE4 cells cultured under polarized conditions release magnetic nanoparticles from the cytosol and into an abluminal compartment without any loss of barrier integrity, when exposed to an external magnetic field [27,44]. As earlier stated, only magnetic nanoparticles and not the entire magnetic OX26 immunoliposomes were detectable in our system. Therefore, we can with certainty state, that the magnetic nanoparticles are detectable outside the vessels only when the magnetic force is applied. We speculate that OX26-liposomes are retained in the BCECs or even recycled into the bloodstream, while the magnetic nanoparticles are dragged out of the OX26-liposomes when subjected to the external magnetic force. These speculations are based on earlier studies stating that anti-transferrin antibodies will be retained in BCECs [7,17,18,20,21,22,23,24,25]. 

To the best of our knowledge, the present study is the first to utilize magnetic OX26 immunoliposomes together with an external magnet; therefore, the results obtained cannot directly be compared to other results targeting nanoparticles conjugated with OX26. Only a few studies have investigated transport across the intact BBB under the influence of an external magnet; e.g., [27,28,33,56,57,58,59,60,61,62,63]. The brain’s uptake of non-conjugated magnetic liposomes has been examined in mice following intravenous injection [33], which supports the present data in that magnetic liposomes may pass the BBB once targeted to BCECs. Carrying out experiments without emptying the brain vessels for particles by vascular perfusion compromises the resolution of whether particles were distributed to intra or paravascular compartments [33]. Magnetic liposomes present intravascularly, probably significantly contribute to the quantitative measurements of BBB transport [33]. Non-conjugated magnetic liposomes were demonstrated perivascularly in the normal and pathological murine brain and indicate that magnetic liposomes may pass the intact BBB once taken up by BCECs without causing significant toxicity [3,28]. The evidence of non-conjugated magnetic liposomes being transported across the BBB are mixed [56,64]. Injecting non-conjugated magnetic immunoliposomes intravenously in mice while monitoring in real-time led to an accumulation of magnetic immunoliposomes trapped within cerebral venules and no evidence to support crossing the BBB [56]. A more recent study indeed, claimed transport across the BBB following the addition of external magnetic fields in the range of 28–80 mT [64], which suggests that the conjugation of magnetic liposomes to a targeting molecule like OX26 or other molecules with affinity for receptors expressed by BCECs are beneficial for the uptake and concentration of magnetic immunoliposomes within BCECs.

Transferrin-conjugated magnetic immunoliposomes in combination with a 0.08 T external magnet increase transmigration trough an intact human in vitro BBB compared to magnetic liposomes without transferrin and the use of magnetic force [58]. Exogenous transferrin may be outcompeted by endogenous transferrin in vivo, whereas OX26 does not compete with the same binding site as endogenous transferrin [65]. Therefore, OX26 would be a better targeting moiety to utilize for targeting the BCECs.

Conjugating lactoferrin to magnetic nanoparticles for targeting the BCECs significantly improved the permeation through cultured primary porcine BCECs [57]. However, the non-conjugated particles, nonetheless, did pass through this BBB model in considerable numbers, indicating less than optimal barrier integrity. In vivo validation of BBB transport sought by intravenous injection and followed by measurements by magnetic resonance imaging (MRI) displayed enhanced contrast in animals that received lactoferrin-coated nanoparticles [57]. MRI does not provide the optimal methodology for the detection of transcytosis across the BBB, and it remains to be seen if such particles pass the BBB or are simply retained inside BCECs.

Without an external magnetic force, there is no experimental evidence for blood to brain delivery of magnetic nanoparticles enveloped in magnetic OX26 immunoliposomes, which concurs with previous studies on OX26-MAbs [7,17,18,20]. Still, uptake of cDNA containing magnetic OX26 immunoliposomes could be relevant for transfection of BCECs with purpose of inducing secretion and release of neurotrophic factors further into the brain [66]. Targeted delivery to BCECs is also highly relevant when dealing with solid tumors by enabling the accumulation of nanoparticles in tumor endothelial cells and inducing their apoptosis [2,3,67].

In conclusion, we show that magnetic OX26 immunoliposomes increase uptake and retention of magnetic nanoparticles in BCECs, and that an external magnetic force can aid the movement of magnetic nanoparticles from BCECs into the brain parenchyma. Our ultrastructural examinations of the magnetic immunoliposomes, however, were unable to confirm complete encapsulation of all naked nanoparticles within liposomes, suggesting that our data could have been derived from naked magnetic nanoparticles being released from liposomes when applied with external magnetic force, calling into question the hypothesis that external magnetic force drags targeted magnetic immunoliposomes through the BBB. While the results imply a strategy for designing targeted magnetic immunoliposomes for specific and increased uptake into BCECs, their design and the strategy for amplification of the external magnetic force needs further improvement.

## Figures and Tables

**Table 1:** Hydrodynamic size, polydispersity index (PDI), and ζ-potential of magnetic nanoparticles, magnetic liposomes, and magnetic OX26 immunoliposomes.

	Hydrodynamic Size (nm)	PDI	ζ-potential (mV)
Magnetic Nanoparticles	117.17 ± 1.17	0.17	−14.83 ± 0.42
Magnetic Liposomes	150.20 ± 2.44	0.22	15.66 ± 1.65
OX26-Magnetic Immunoliposomes	181.90 ± 6.32	0.29	−7.14 ± 3.13

## Supplementary Materials

The following are available online at https://www.mdpi.com/1996-1944/12/21/3576/s1. Figure S1: Transmission electron microscopy (TEM) of magnetic nanoparticles and magnetic OX26 immunoliposomes; Figure S2: The effects of prolonged incubation time and fixation with paraformaldehyde on the distribution of magnetic liposomes.

## Abbreviations

BBB, blood brain barrier; BCECs, brain capillary endothelial cells; BSA, bovine serum albumin; DAPI, 4’,6-diamidino-2-phenyindole; HEPES, 4-(2-hydroxyethyl)-1-piperazineethanesulfonic acid; OX26, monoclonal antibody against the transferrin receptor; OX26-MAbs, OX26 monoclonal antibodies; P, postnatal; PEG, polyethylene glycol; RBE4, rat brain endothelial cells; SATA, *N*-succinimidyl-*S*-acetylthioacetate.
