# Supplementary material for: Evaluation of Targeted Delivery to the Brain Using Magnetic Immunoliposomes and Magnetic Force
Source: Materials (Basel). 2019 Oct 31;12(21):3576. doi: 10.3390/ma12213576 (PMC6861967; doi:10.3390/ma12213576)
Supplement: Supplementary file 1 [file materials-12-03576-s001.pdf]

materials

Article

Evaluation of Targeted Delivery to the Brain Using Magnetic Immunoliposomes and Magnetic Force

Louiza Bohn Thomsen [1,*,†], Thomas Linemann [1,†], Svend Birkelund [2], Gitte Abildgaard Tarp [1] and Torben Moos [1,*]

[1] Laboratory of Neurobiology, Biomedicine Group, Department of Health Science and Technology, Aalborg University, 9220 Aalborg East, Denmark; tl@linemann.com (T.L.); Gitte.A.Arendt@hotmail.com (G.A.T.)
[2] Laboratory of Medical Mass Spectrometry, Biomedicine Group, Department of Health Science and Technology, Aalborg University, 9220 Aalborg East, Denmark; sbirkelund@hst.aau.dk
* Correspondence: E-mail: lbt@hst.aau.dk (L.B.T.); tmoos@hst.aau.dk (T.M.); Tel.: +45-99407878 (L.B.T.)
† These authors contributed equally to this work

Supplementary materials

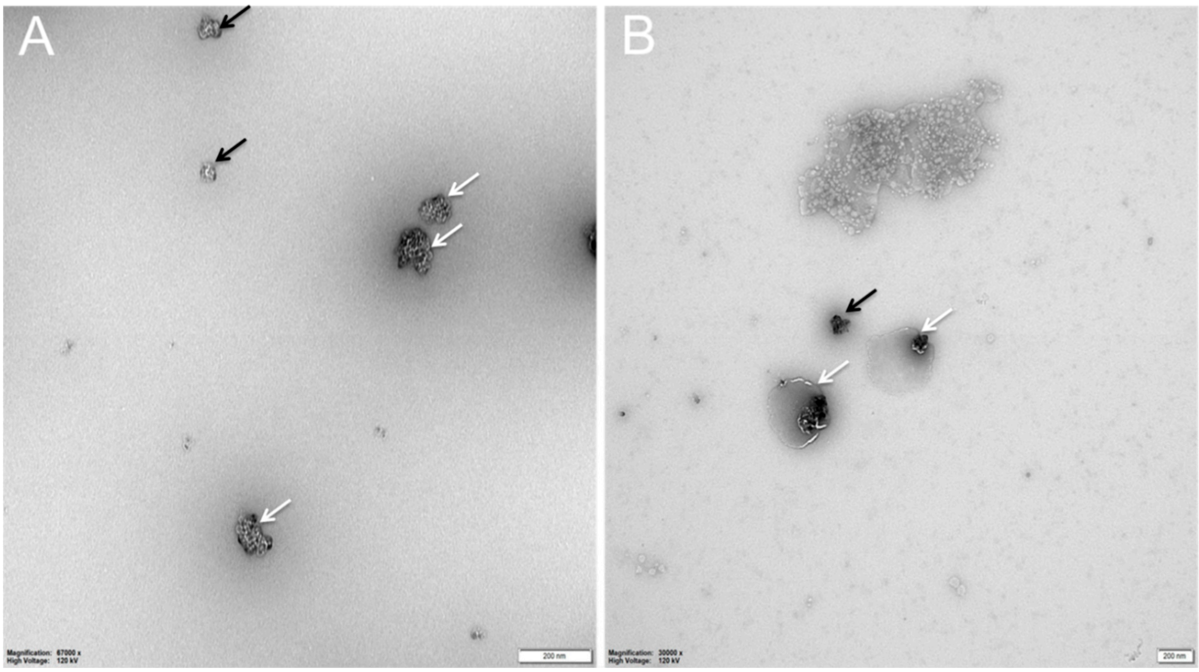

Figure S1. Transmission electron microscopy (TEM) of magnetic nanoparticles and OX26-Magnetic immunoliposomes. (**A**) Magnetic nanoparticles. Many of the magnetic nanoparticles were consistently size around 117nm as obtained using DLS (white arrows). Smaller magnetic nanoparticles could also be observed (black arrows) (A). (**B**) OX26-Magnetic immunoliposomes. Magnetic nanoparticles encapsulated in OX26-magnetic immunoliposomes were observed (white arrows). The OX26- magnetic immunoliposomes were mostly observed in a size of 3-400 nm in diameter, but also smaller and larger OX26- magnetic immunoliposomes were observed ranging from approximately 200nm to 500nm. Furthermore a fraction of both un-encapsulated magnetic nanoparticles (black arrows) and OX26-liposomes without magnetic nanoparticles encapsulated was observed. A few OX26-magnetic immunoliposomes had two magnetic nanoparticles embedded, as can be seen in the lower left OX26-magnetic immunoliposomes on B. Scale bar on both A and B = 200 μm.

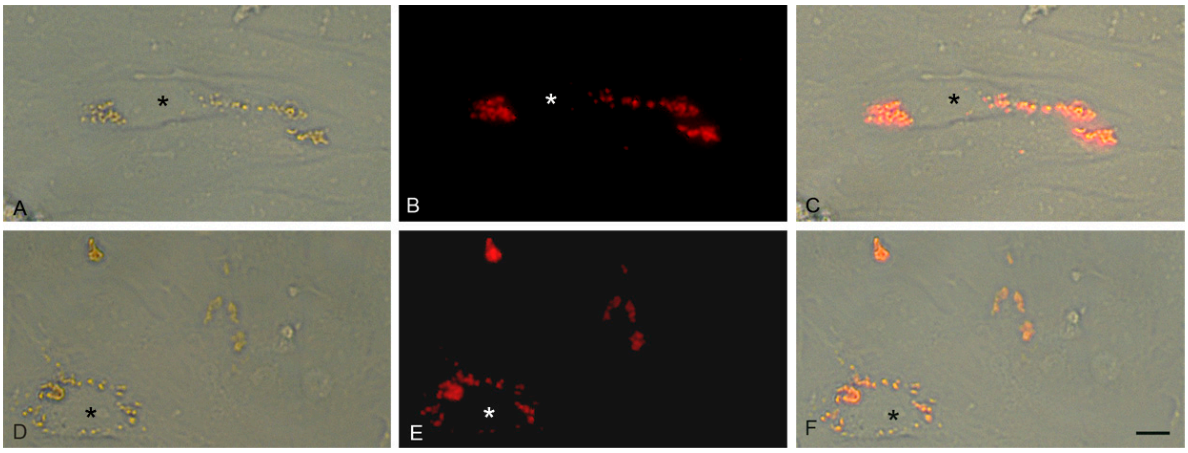

Figure 2. The effects of prolonged incubation time and fixation with paraformaldehyde on the distribution of magnetic liposomes magnetic liposomes. RBE4 cells were incubated with magnetic liposomes for 48 hrs. The cells were exposed for the magnetic liposomes this time interval to examine whether this would change the subcellular distribution of the magnetic liposomes, which could indicate that the magnetic liposomes had been degraded within the cells. However, the distribution of magnetic liposomes was indistinguishable when comparing with cells incubated with shorter time intervals. Likewise, the distribution of magnetic liposomes and their corresponding fluorescence were indistinguishable when comparing the cells before and after fixation. (**A**–**C**) Upper row shows a single RBE4 cell that did not receive fixation. (**A**) Phase-contrast image in where the magnetic particles are seen as yellow dots. (**B**) Red fluorescence of the same cell. (**C**) Overlay. The BCEC nucleus is identified by an asterisk. (**D**–**F**) Lower row shows a single RBE4 cell that received fixation with 4% paraformaldehyde for 15 min. (**D**) Phase-contrast image in where the magnetic particles are seen as yellow dots. (**E**) Red fluorescence of the same cell. (**F**) Overlay. The cellular nucleus is identified by an asterisk. Scale bar = 5 μm.
